# Genetic diversity and population structure of trifoliate yam (*Dioscorea dumetorum* Kunth) in Cameroon revealed by genotyping-by-sequencing (GBS)

**DOI:** 10.1186/s12870-018-1593-x

**Published:** 2018-12-18

**Authors:** Christian Siadjeu, Eike Mayland-Quellhorst, Dirk C. Albach

**Affiliations:** Institute for Biology and Environmental Sciences, Biodiversity and Evolution of Plants, Carl-von-Ossietzky University Oldenburg, Carl-von-Ossietzky Str. 9-11, 26111 Oldenburg, Germany

**Keywords:** Cameroon, *D. dumetorum*, Yam, Genetic diversity, Population structure, GBS, Ploidy

## Abstract

**Background:**

Yams (*Dioscorea* spp.) are economically important food for millions of people in the humid and sub-humid tropics. *Dioscorea dumetorum* (Kunth) is the most nutritious among the eight-yam species, commonly grown and consumed in West and Central Africa. Despite these qualities, the storage ability of *D. dumetorum* is restricted by severe postharvest hardening of the tubers that can be addressed through concerted breeding efforts. The first step of any breeding program is bound to the study of genetic diversity. In this study, we used the Genotyping-By-Sequencing of Single Nucleotide Polymorphism (GBS-SNP) to investigate the genetic diversity and population structure of 44 accessions of *D. dumetorum* in Cameroon. Ploidy was inferred using flow cytometry and gbs2ploidy.

**Results:**

We obtained on average 6371 loci having at least information for 75% accessions. Based on 6457 unlinked SNPs, our results demonstrate that *D. dumetorum* is structured into four populations. We clearly identified, a western/north-western, a western, and south-western populations, suggesting that altitude and farmers-consumers preference are the decisive factors for differential adaptation and separation of these populations. Bayesian and neighbor-joining clustering detected the highest genetic variability in *D. dumetorum* accessions from the south-western region. This variation is likely due to larger breeding efforts in the region as shown by gene flow between *D. dumetorum* accessions from the south-western region inferred by maximum likelihood. Ploidy analysis revealed diploid and triploid levels in *D. dumetorum* accessions with mostly diploid accessions (77%). Male and female accessions were mostly triploid (75%) and diploid (69%), respectively. The 1C genome size values of *D. dumetorum* accessions were on average 0.333 ± 0.009 pg and 0.519 ± 0.004 pg for diploids and triploids, respectively.

**Conclusions:**

Germplasm characterization, population structure and ploidy are an essential basic information in a breeding program as well as for conservation of intraspecific diversity. Thus, results obtained in this study provide valuable information for the improvement and conservation of *D. dumetorum*. Moreover, GBS appears as an efficient powerful tool to detect intraspecific variation.

**Electronic supplementary material:**

The online version of this article (10.1186/s12870-018-1593-x) contains supplementary material, which is available to authorized users.

## Background

Yams (*Dioscorea* spp.) constitute a staple food for over 300 million people in the humid and sub-humid tropics. About 600 species are described and are widely distributed throughout the tropics [1]. *Dioscorea dumetorum* has the highest nutrient value among eight other yam species commonly grown and consumed in West and Central Africa [2]. The species originated in tropical Africa and occurs in both wild and cultivated forms. Its cultivation is restricted to West and Central Africa [3], and widespread in western Cameroon. Tubers of *D. dumetorum* are protein-rich (9.6%) with fairly balanced essential amino acids and its starch is easily digestible [4–6]. Agronomically, *D. dumetorum* is high-yielding, with yield of 40 tons/hectare recorded in agricultural stations [7]. *Dioscorea dumetorum* is also recognized for its pharmaceutical properties. A novel bioactive compound dioscoretine has been identified in *D. dumetorum* [8], which can be used advantageously as a hypoglycemic agent in anti-diabetic medications [9].

Despite these qualities, the storage ability of *D. dumetorum* is restricted by severe postharvest hardening of the tubers, which begins within 24 h after harvest and renders them unsuitable for human consumption [2]. According to Treche and Delpeuch [10], the usual storage conditions in West Africa (under airy warehouse, shelter from sunlight) induce 100% losses after 4 months of storage. It is manifested by the loss of culinary quality due to a combination of factors resulting from normal but inadvertently deleterious reactions leading to textural changes [11]. Therefore, *D. dumetorum* is consumed exclusively during its limited harvest period and only freshly harvested tubers are cooked and sold to consumers. To add more value to *D. dumetorum* as an important source of food and energy, hardened tubers are transformed into instant flour [12]. However, flour obtained directly from hardened tubers has poor organoleptic qualities, such as coarseness in the mouth [4]. Thus, other techniques have been used, such as salt soaking treatment [13] and fermentation [14], but the hardening phenomenon has not been surmounted. Consequently, molecular breeding of *D. dumetorum* appears as the appropriate method to overcome this phenomenon.

The study of genetic diversity is an important, early step in plant breeding. Highlighting this variability is part of the characterization of germplasm under investigation. In our recent study on the phenotypic diversity of *D. dumetorum* we found relatively high diversity of morphological characters suggesting high underlying genetic diversity [15]. Indeed, the expression of morphological characters are subject to agro-climatic variations and thus provides limited genetic information. Therefore, molecular markers that are not subject to environmental variations are necessary for estimation of genetic diversity. The development of molecular markers over the last 30 years has enabled the study of diversity and evolution as well as germplasm characterization [16]. Among these markers, Single Nucleotide Polymorphisms (SNPs) have emerged as the most widely used genotyping markers due to their abundance in the genome allowing not only germplasm characterization but also quantification of relative proportions of ancestry derived from various founder genotypes of currently grown cultivars [16]. Moreover, the development of traditional markers like SSRs, RFLPs and AFLPs was a costly, iterative process that involved either time-consuming cloning and enzyme testing or primer design steps that could not easily be parallelized [17].

Genotyping-By-Sequencing (GBS) has emerged as a new approach to mitigate these constraints. The method has been demonstrated to be suitable for population studies, germplasm characterization, genetic improvement, trait mapping in a variety of diverse organisms and thereby, SNP discovery and genotyping of multiple individuals are performed cost-effectively and efficiently [18]. GBS is performed by an initial digest of sample DNA with restriction enzymes reducing genome complexity followed by a round of PCR to generate a high-throughput sequencing library [19]. Reducing genome complexity with restriction enzymes is quick, extremely specific and highly reproducible [19]. Unlike other similar approaches using restriction enzymes, GBS is technically simple [20]. Besides, bioinformatic pipelines are publicly available [21] and GBS can be easily applied to non-model species with limited genomic information [20]. This method has been successfully used on Cassava (*Manihot esculenta* Crantz) [22], guinea yam [23] and water yam [24], which demonstrated the power of GBS-SNP genotyping as a suitable technology for high-throughput genotyping in yam.

Genetics of yams is least understood and remains largely neglected among the major staple food crops due to several biological constraints and research neglect [25]. Some progress has been made in germplasm characterization and the development of molecular markers for genome analysis. Various dominant molecular markers (AFLP, RAPD) have been used on yam with little success (e.g., [9]). Additionally, genomic microsatellite markers have been developed for yam species [24–32]. However, no markers have been developed for *D. dumetorum* and its genetics is the least known in spite of its qualities among the cultivated yam. Until now, no information is available using SNP genotyping to assess population structure, genetic diversity and the relationship among *D. dumetorum* cultivars.

A possible additional factor that influences population structure and genetic diversity is polyploidy. Polyploidy has several advantages for plant breeding such as the increment in plant organs (“gigas”effect), buffering of deleterious mutations, increased heterozygosity, and heterosis (hybrid vigor) [33]. In yam, ploidy increase is correlated with growth vigor, higher and more stable tuber yield and increased tolerance to abiotic and biotic stress [33, 34]. Recent studies using flow cytometry revealed diploid and triploid levels in *D. dumetorum* with predominance of the diploid cytotype [35, 36]. Therefore, the objective of this study is to understand the genetic diversity and the population structure of *D. dumetorum* using the genotyping-by-sequencing (GBS) in relation to ploidy information.

## Methods

### Plants materials

**Fig. 1:**
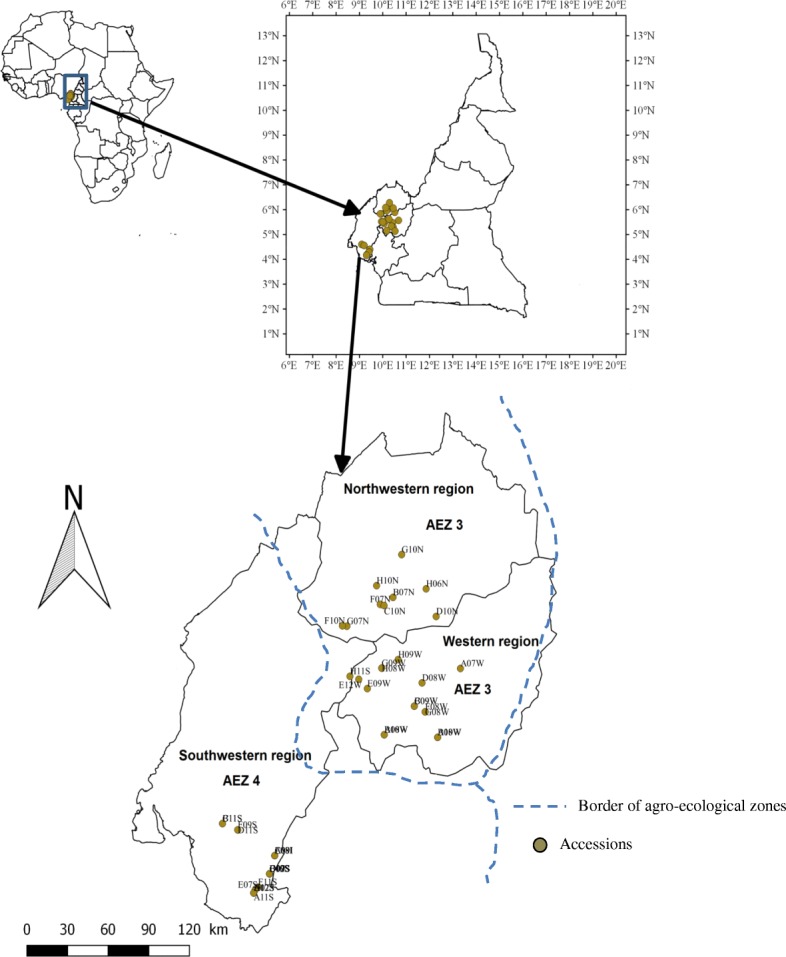
Sampling map of *D. dumetorum* accessions in Cameroon. Boundaries of agro-ecological zones (AEZ) were defined according to [38]

**Fig. 2:**
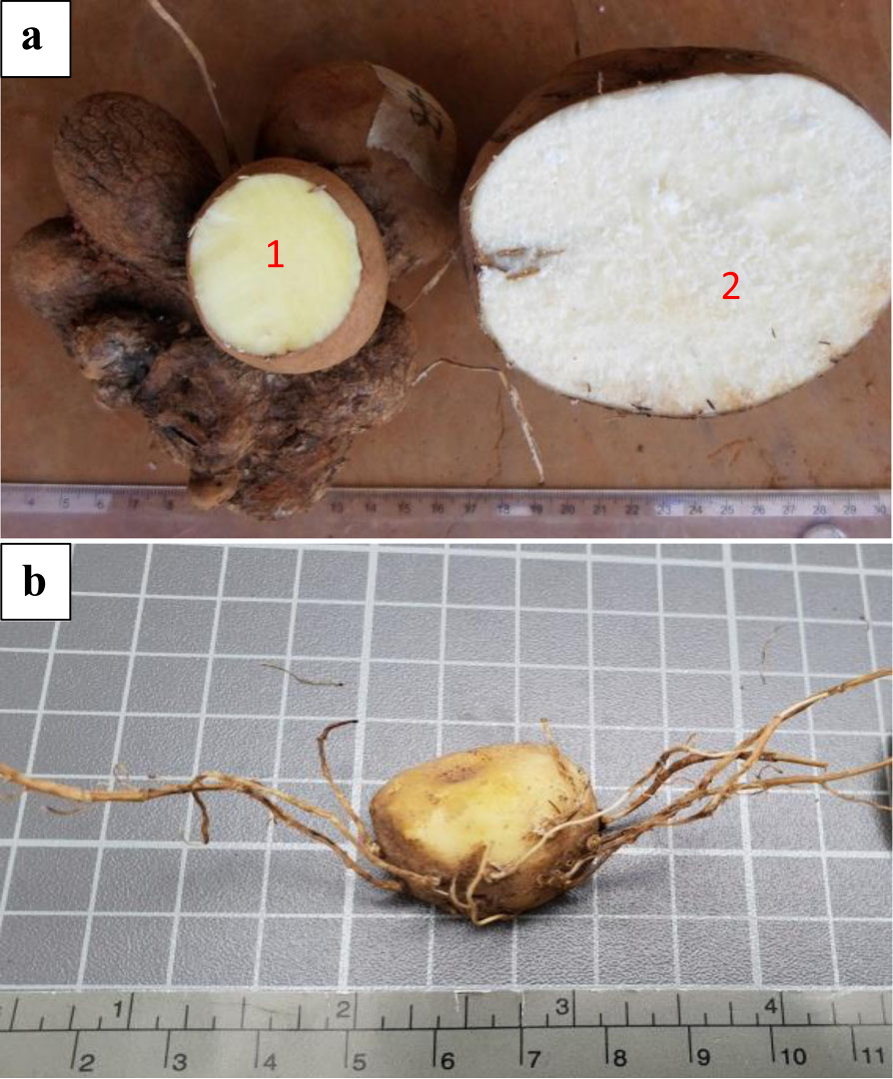
*D. dumetorum* tuber forms. **1a**) Accession with few roots on the tuber and yellow flesh colour. **2a**) accession with few roots on the tubers and white flesh colour **b**) accession with many roots on the tuber

Overall, 44 accessions of *D. dumetorum* were used in this study (Table 1). All these accessions were collected from different localities in the major yam growing regions (western, south-western, and north-western) of Cameroon, with an additional three accessions of *D. dumetorum* from Nigeria complementing the dataset (Fig. 1). Western and north-western regions belong to agro-ecological zone (AEZ) 3 and the south-western region to AEZ 4 of Cameroon [38]. Most of these accessions were previously used for morphological characterization [15] and hardness assessment [39]. Here, we selected some characters related to tubers (Fig. 2).The yam tubers of these accessions were planted in April 2015 at the “Ferme Ecole de Bokué” in the western region of Cameroon (latitude 05°20.040’ N and longitude 010°22.572 E). Silica-dried young leaves were transported to Oldenburg (Germany) for molecular analyses. Genomic DNA was extracted using an innuPREP Plant DNA kit (Analytik Jena, Jena, Germany).

**Table 1:** Characteristics of *D. dumetorum* accessions used in this study. * Area belongs to agro-ecological zone 3, ** to agro-ecological zone 4

Code	Accession name	Geographic origin	Roots on the tuber surface	Tuber flesh color	Tuber hardening	Altitude (m)
A07W	Baigon 1	West*	Few	Yellow	Yes	1120
A08W	Fonkouankem 1	West*	Many	Yellow	Yes	1167
A09I	Ibo sweet 3	Nigeria	Few	Yellow	No	56
A10W	Bangangté 1	West*	Few	Yellow	Yes	1350
A11S	Muyuka 1	Southwest**	Few	White	Yes	554
A12S	Muyuka 5	Southwest**	Few	White	Yes	554
B07N	Bambui 1	Northwest*	Few	Yellow	Yes	1262
B08W	Bangou 1	West*	Many	Yellow	Yes	1350
B09W	Bamendjou 2	West*	Many	Yellow	Yes	1647
B10W	Bana 1	West*	Few	Yellow	Yes	1167
B11S	Bekora 1	Southwest**	Few	White	Yes	60
B12S	Country yam 2	Southwest**	Few	White	Yes	62
C07S	Banga bakundu sweet 1	Southwest**	Few	White	Yes	62
C08I	Ibo sweet 2	Nigeria	Many	White	Yes	56
C09W	Bamendjou 1	West*	Few	Yellow	Yes	1647
C10N	Nkwen 1	Northwest*	Few	Yellow	Yes	1251
C11S	Bekora 2	Southwest**	Few	White	Yes	60
C12S	Muyuka 3a	Southwest**	Few	White	Yes	62
D07S	Country yam 1	Southwest**	Few	White	Yes	62
D08W	Bamokonbou 1	West*	Many	Yellow	Yes	1414
D09S	Muyuka 3	Southwest**	Few	White	Yes	62
D10N	Bambalang 1	Northwest*	Few	Yellow	Yes	1185
D11S	Mabondji sweet yellow 1	Southwest**	Few	Yellow	Yes	80
D12S	Penda-boko sweet 1	Southwest**	Few	White	Yes	554
E07S	Lysoka sweet 1	Southwest**	Many	Yellow	Yes	60
E08I	Ibo sweet 1	Nigeria	Few	White	Yes	56
E09W	Dschang 1	West*	Few	Yellow	Yes	1337
E10S	Mbongue sweet 1	Southwest**	Few	White	Yes	62
E12W	Fong-tongo 1	West*	Many	Yellow	Yes	1460
F07 N	Mankon 1	Northwest*	Few	Yellow	Yes	1253
F08 W	Bayangam 1	West*	Few	Yellow	Yes	1560
F09S	Mabondji sweet white 1	Southwest**	Few	White	Yes	80
F10 N	Batibo 1	Northwest*	Few	Yellow	Yes	1127
F11S	Buea sweet white yam 1	Southwest**	Few	White	Yes	438
G07 N	Guzang 1	Northwest*	Many	Yellow	Yes	1233
G08 W	Bayangam 2	West*	Many	Yellow	Yes	1560
G09 W	Bangang 1	West*	Few	Yellow	Yes	1776
G10 N	Fundong 1	Northwest*	Few	Yellow	Yes	1554
H06N	Babungo 1	Northwest*	Few	Yellow	Yes	1182
H07S	Buea sweet 1	Southwest**	Many	Yellow	Yes	554
H08W	Bangang 2	West*	Many	Yellow	Yes	1776
H09W	Babadjou 1	West*	Few	Yellow	Yes	1395
H10N	Bafut 1	Northwest*	Few	Yellow	Yes	1123
H11S	Alou 1	Southwest**	Few	Yellow	Yes	1606

### Preparation of libraries for next-generation sequencing

A total of 200 ng of genomic DNA for each sample were digested with 1 Unit MslI (New England Biolabs, NEB) in 1x NEB4 buffer in 30 μl volume for 1 h at 37 °C. The restriction enzyme was heat inactivated by incubation at 80 °C for 20 min. Afterwards, 15 μl of digested DNA were transferred to a new 96well PCR plate, mixed and stored on ice first with 3 μl of one of the 192 L2 ligation adaptors (Ovation Rapid DR Multiplex System, Nugen Technologies, Leek, The Netherlands) and then with 12 μl master mix (combination of 4.6 μl D1 water/ 6 μl L1 ligation buffer mix/ 1.5 μl L3 ligation enzyme mix). Ligation reactions were incubated at 25 °C for 15 min followed by inactivation of the enzyme at 65 °C for 10 min. Then, 20 μl of the kits ‘final repair’ master mix were added to each tube and the reaction was incubated at 72 °C for 3 min. For library purification, reactions were diluted with 50 μl TE 10/50 (10 mM Tris/HCl, 50 mM EDTA, pH:8.0) and mixed with 80 μl magnetic beads, incubated for 10 min at room temperature and placed for 5 min on a magnet to collect the beads. The supernatant was discarded and the beads were washed two times with 200 μl 80% Ethanol. Beads were air-dried for 10 min and libraries were eluted in 20 μl Tris Buffer (5 mM Tris/HCl, pH 9). Each of the 45 libraries (including one technical repeat) were amplified with 10 μl of the purified restriction product in 20 μl PCR reactions using 4 μl MyTaq (Bioline) 5x buffer, 0.2 μl polymerase and 1 μl (10 pmol/μl) of standard Illumina TrueSeq amplification primers. Cycle number was limited to ten cycles. Then, 5 μl from each of the 48 amplified libraries were pooled. PCR primers and small amplicons were removed by magnetic bead purification using 0.6 volume of beads. The PCR polymerase was removed by an additional purification on Qiagen MinElute Columns. The pooled library was eluted in a final volume of 20 μl Tris buffer (5 mM Tris/HCl, pH 9). The final library pool was sent to LGC genomics (Berlin, Germany) and sequenced on an Illumina NextSeq with 1.5 million 150 bp paired-end reads for each sample. Additional steps at LGC for the sequencing preparation were normalization, reamplification and size selection. Normalization was conducted using Trimmer Kit (Evrogen). For this 1 μg pooled GBS library in 12 μl was mixed with 4 μl 4x hybridization buffer, denatured for 3 min at 98 °C and incubated for 5 h at 68 °C to allow re-association of DNA fragments. 20 μl of 2 x DSN master buffer was added and the samples were incubated for 10 min at 68 °C. One unit of DSN enzyme (1 U/μl) was added and the reaction was incubated for another 30 min. The reaction was terminated by the addition of 20 μl DSN Stop Solution, purified on a Qiagen MinElute Column and eluted in 10 μl Tris Buffer (5 mM Tris/HCl pH 9).The normalized library pools were re-amplified in 100 μl PCR reactions using MyTaq (Bioline). Primer i5-Adaptors were used to include i5-indices into the libraries, allowing parallel sequencing of multiple libraries on the Illumina NextSeq 500 sequencer. Cycle number was limited to 14 cycles. The nGBS libraries were size selected using Blue Pippin, followed by a second size selection on a LMP-Agarose gel, removing fragments smaller than 300 bp and those larger than 400 bp. Libraries were sequenced on an Illumina NextSeq 500 using Illumina V2 Chemistry.

### GBS data analysis

GBS data were analyzed using the custom software pipeline iPyrad (Versions: 0.7.19 and 0.7.28) developed by Eaton and Ree [21] for population genetic and phylogenetic studies. It includes seven steps to demultiplex and quality filtering, cluster loci with consensus alignments and SNP calling with SNP filtering to the final SNP matrix, which can be transferred into various output formats. We have conducted demultiplexing and QC separately to retrieve fastq sequences as input for iPyrad. The restriction sites and barcodes were trimmed for each sequence, bases with a quality score less than PHRED 20 were changed into N and sequences having more than 5% of N were discarded. Step 3 of iPyrad used in our de-novo SNP analysis VSEARCH [40] for dereplication and merging of paired reads and for clustering reads per sample into putative loci with 85% sequence similarity. Alignments of consensus sequences of the putative loci were built with MUSCLE [41]. After estimation of sequencing errors (Π) and heterozygosity (ɛ), consensus alleles were estimated with these estimated parameters and the number of alleles was recorded. Resulting consensus alleles were again clustered with VSEARCH and aligned with MUSCLE. Base SNPs were called when loci were observed in at least 75% of the samples, had not more than 20 SNPs and eight indels and heterozygous sites in 50% of the samples, but all samples were treated as diploid, thus allowing two haplotypes per polymorphic site.

### Phylogenetic inference

An unrooted tree was generated using the neighbor-net method in SplitsTree (Version 4.14.6) [42] based on the concatenated GBS data. To control whether or not the introduction of triploid accessions affected our phylogenetic analysis, we constructed dendrograms with and without triploid accessions.

### Historical relationship between accessions (TreeMix)

Historical relationships between *D. dumetorum* accessions including possible gene flow events was assessed through the maximum likelihood method implemented in TreeMix (version 1.13) [43]. TreeMix reconstructs the possible migrations between populations based on allele frequency of genomic data. It uses a method that allows for both population splits and gene flow. We defined the population parameter as 0, because we worked at the individual level. Of 25,541 SNPs loci investigated, 157 SNPs were filtered to get a gap free matrix and used to determine the relationships between the accessions. The tree was built with the confidence of 1000 bootstrap replicates and visualized with toytree (version 0.1.4) and toyplot (version 0.16.0).

### Population structure analysis

Analysis of population structure was performed using the software STRUCTURE [44] and MavericK [45]. Structure uses a Bayesian model-based clustering method with a heuristic approach for estimation whereas MavericK uses a computation technique called Thermodynamic Integration (TI). However, the mixture modeling framework is identical in both programs [45]. The analysis was carried out in STRUCTURE using the admixture model across 10 replicates (K of 2 to 5) of sampled unlinked SNPs (one randomly chosen SNP per ipyrad-cluster). A burn-in period of 10,000 iterations and 100,000 Markov Chain Monte Carlo (MCMC) replicates were run. The true number of clusters (K) was detected using Evanno’s method [46] implemented in STRUCTURE HARVESTER [47]. The MCMC implementation of MavericK differs slightly, although the core model assumed is identical to that used in Structure [45]. Thus, the admixture model across five replicates (K of 2 to 5) was run with a burn-in period of 2000 iterations and 10,000 MCMC. The best value of K was detected in 25 TI rungs each for a range of K (2 to 5) with default settings.

### Ploidy/genome size estimation

For each accession, about 1 cm^2^ of young leaf was co-chopped with a standard using a razor blade in a Petri dish containing 1.1 mL ice-cold Otto I buffer (0.1 M citric acid monohydrate and 5% Triton X-100). We used *Solanum lycopersicum* L. ‘Stupicke’ (1C = 0.98 pg; [48] as the internal standard. The chopped material and buffer were then filtered through a Cell-Tric 30-μm filter into a plastic tube, and 50 μL RNase were added. After incubation in a water bath for 30 min at 37 °C, 450 μL of the solution were transferred to another tube, to which 2 mL Otto II (propidium iodide + Na_2_HPO_4_) were added. This solution was placed at 4 °C for 1 h. The samples were analyzed using a CyFlow flow cytometer (Partec GmbH, Münster, Germany). For each accession, three replicates comprising 5000 counts were measured. We measured the genome size of 17 out 44 *D. dumetorum* accessions due to the loss of certain accessions, in which the sex has been identified. Ploidy level of the remaining accessions (27) was assessed using the R package gbs2ploidy [49]. This method infers cytotypes based on the allelic ratios of heterozygous SNPs identified during variant calling within each individual. Data was prepared by acquiring a *.vcf output file for all specimens from iPyrad using VCFConverter2.py (https://github.com/dandewaters/VCF-File-Converter) as in [50]. Cytotypes were estimated in two ways: 1) without reference to accessions of known ploidy and 2) with reference of 17 accessions for which ploidy is known) from flow cytometry as set of triploids and diploids to the 27 remaining accessions.

## Results

### GBS data analysis summary

We generated an average of 2.2 million raw reads per *D. dumetorum* accessions by Illumina sequencing (Table 2). After filtering we obtained on average 1.3 × 10^4^ reads clustered at 85%, with an average depth per accession of 53. The maximum likelihood average estimate of heterozygosity (ɛ = 1.1 × 10^− 2^) was greater than the sequence error rate (Π = 6 × 10^− 3^). Consensus sequences were called for each cluster, yielding on average 32,532 reads per accession. We recorded on average 6371 loci recovered in at least 75% of accessions. Accession D09S had a markedly higher proportion of missing data.

**Table 2:** Summary statistics of filtering and clustering GBS data from *D. dumetorum*

Accessions code	Raw reads	clusters total at 85%	Heterozygosity (ɛ)	Sequence error rate (Π)	Consensus reads	Loci with 75% of samples	Mean depth
A07W	97,6092	85,513	0.01	0.006	23,641	6411	22.03
A08W	4,208,284	188,712	0.013	0.006	44,753	6926	93.15
A09I	6,850,560	230,744	0.016	0.006	50,290	6710	153.87
A10W	1,403,568	105,485	0.01	0.006	29,316	6806	32.24
A11S	2,355,324	153,699	0.012	0.007	36,254	6797	50.5
A12S	2,224,370	194,901	0.012	0.007	39,005	6599	38.43
B07N	6,315,512	270,497	0.015	0.006	50,836	6776	132.11
B08W	3,224,481	152,315	0.011	0.006	38,488	6938	75.73
B09W	4,821,443	227,297	0.013	0.006	49,469	6873	107.54
B10W	2,246,679	123,018	0.011	0.006	32,260	6978	54.09
B11S	1,675,059	114,487	0.011	0.007	31,882	6589	35.9
B12S	2,823,683	140,521	0.012	0.006	37,261	6843	64.72
C07S	10,482,599	328,036	0.016	0.006	65,451	6621	219.94
C08I	1,051,320	100,553	0.01	0.007	25,978	6340	23.27
C09W	3,945,117	180,341	0.013	0.006	42,150	6935	91.99
C10N	1,060,648	94,092	0.01	0.007	25,254	6592	24.77
C11S	1,937,689	125,788	0.012	0.006	31,880	6815	43.75
C12S	479,412	60,756	0.009	0.007	16,586	4535	10.32
D07S	3,137,681	162,346	0.012	0.006	38,116	6809	72.13
D08W	1,127,041	85,710	0.01	0.006	25,476	6491	26.71
D09S	24,555	8580	0.012	0.006	883	208	0.2
D10N	814,774	77,508	0.01	0.006	22,223	6298	19.69
D11S	2,860,962	166,597	0.012	0.006	41,004	6818	60.81
D12S	476,212	58,172	0.011	0.007	16,603	4777	11.11
E07S	310,575	49,544	0.01	0.007	11,810	3427	5.73
E08I	2,329,405	156,765	0.011	0.006	36,697	6783	52.83
E09W	1,182,619	119,258	0.01	0.006	27,799	6354	23.22
E10S	1361,644	110,821	0.012	0.007	28,732	6665	28.6
E12W	701,654	79,193	0.01	0.006	21,530	5765	15.23
F07 N	2,413,712	138,285	0.011	0.006	33,944	6969	56.7
F08 W	1,735,184	114,265	0.011	0.006	30,603	6665	40.36
F09S	1,643,257	110,155	0.011	0.007	30,166	6733	37.7
F10 N	739,588	68,577	0.01	0.006	20,579	5869	16.84
F11S	1,542,460	98,117	0.01	0.006	28,884	6651	36.72
G07 N	3,845,340	171,087	0.013	0.006	40,668	6924	88.43
G08 W	1,637,683	115,971	0.011	0.006	31,144	6917	38.44
G09 W	675,273	68,986	0.01	0.006	20,229	5793	15.56
G10 N	2,277,786	128,426	0.011	0.006	32,260	6955	54.21
H06N	4,854,717	207,191	0.014	0.006	49,949	6919	107.4
H07S	3,731,409	192,342	0.012	0.006	46,340	6790	76.41
H08W	3,057,515	146,899	0.012	0.006	35,916	6936	71.65
H09W	1,579,956	107,146	0.01	0.006	28,594	6951	39.04
H10N	1,920,829	109,146	0.011	0.006	31,018	6942	46.4
H11S	1,530,490	103,703	0.01	0.006	29,508	6831	34.52
Mean	2,399,867	132,535	0.011	0.006	32,532	6371	53.43

### Phylogenetic inference

**Fig. 3:**
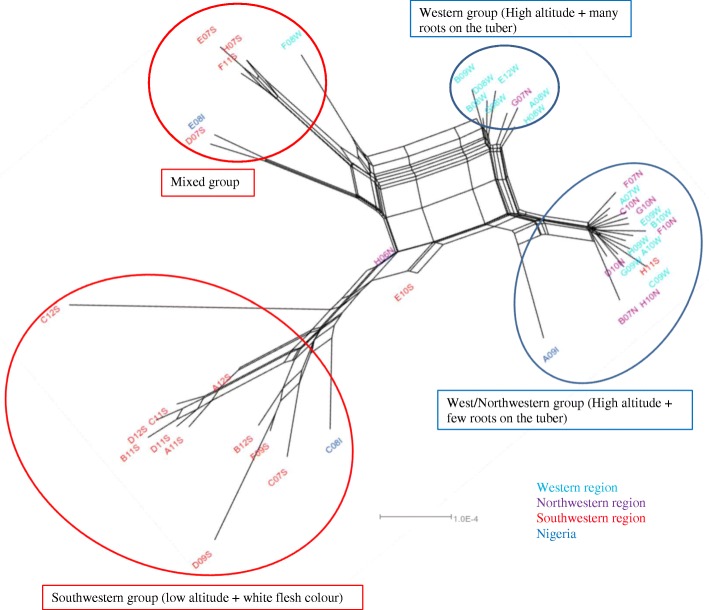
Phylogenetic relationships within *D. dumetorum* based on multilocus concatenated SNP sequences alignment from GBS data of 44 accessions

The unrooted neighbor-net clustered the 44 accessions of *D. dumetorum* into four groups: a western/north-western group, a western group, a southwestern group and a mixed group (Fig. 3). However, two accessions (E10S and H06N) were not clustered in these groups. Triploid accessions did not affect the topology of the network (Additional file 1: Figure S1).

The western/north-western group had 16 accessions, 88% were from the western and north-western regions (50% were from West and 50% from North-west). Remaining accessions (12%) were from the southwestern region (H11S) and Nigeria (A09I). In this group, accessions are characterized by yellow flesh color with few roots on the tuber and were from high altitude regions except A09I. Here, all the accessions hardened after harvest except A09I from Nigeria.

The western group consisted of eight accessions; almost all were from the western region and one from the north-western region (G07 N). This group was constituted by accessions with yellow flesh color and many roots on the tuber. They originate all from high altitude regions and hardened after harvest. The western group was closely related to the western/north-western group and differed in the number of roots on the tubers.

The south-western group had 12 accessions originating from the south-western region except C08I from Nigeria. Unlike the western/north-western group and western group, all accessions were from low altitude regions and had white flesh color. However, all accessions hardened after harvest. The fourth group was a mixed group consisting of six accessions, among which four were from the Southwest, one from the West (F08 W) and one from Nigeria (E08I). As compared to the others, the group is variable with respect to tuber characters. Here again, all accessions hardened after harvest.

### Population structure

**Fig. 4:**
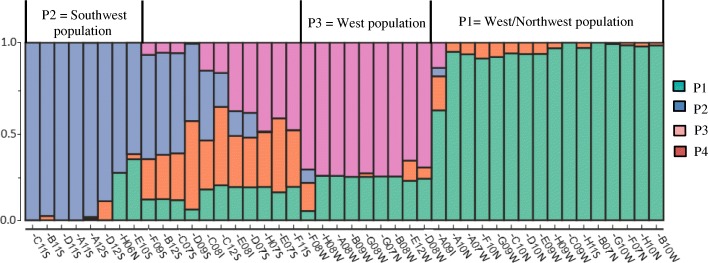
STRUCTURE plot of 44 accessions of *D. dumetorum* with K = 4 clusters based on 6457 unlinked SNPs. Each accession is represented by a single row, which is partitioned into colored segments in proportion to the estimated membership in the three subpopulations

We determined the population structure of *D. dumetorum* using both a Bayesian approach and Thermodynamic Integration (TI) as implemented in STRUCTURE and MavericK, respectively. The STRUCTURE and MavericK results revealed that *D. dumetorum* accessions can be clustered into populations. The delta K (∆K) of Evanno’s method and TI estimator of the evidence for K showed strong peaks at K = 4 and K = 2 respectively (Additional file 2: Figure S2). The K value (K = 4) is the most likely number of populations (Fig. 4), because the existence of four groups was also supported by the neighbor-net method (Fig. 3). In total, 33 accessions (75%) were assigned to one of the first three populations with at least 60% of their inferred ancestry derived from one of the three populations. No accession was assigned to the fourth population with at least 60 of the inferred ancestry. The populations P1, P2 and P3 contained 16, 8, and 9 accessions respectively. The remaining accessions (11) were the result of admixture between the populations.

In population P1, accessions were from the western and north-west region except accessions A09I (Nigeria) and H11S (south-western region). Here, three accessions were assigned 100% to P1, twelve as admixture between P1 and P4 and one accession A09I as admixture of P1xP2xP3xP4. In contrast, all the accessions of population P2 were from the south-western region except H06N (North-west). Four accessions were assigned 100% to P2, two accessions as admixture P2xP4, while two each as admixture P1xP2xP4 and P1xP2. Regarding P3, almost all accessions (8) were from the western region except G07 N from the north-western region. Conversely, no accession was assigned 100% to P3. Five were assigned as admixture P1xP3, three classified as P1xP2xP3 and one as P1xP2xP3xP4. Moreover, the population structure did not change with the increased values of K = 5 (Additional file 3: Figure S3). Comparing results from the STRUCTURE analysis with the neighbor-net, we found generally similar results. Thus, P1 corresponds to the west/north-western population, P2 to the south-western population, and P3 to the western population. No accessions belonging to P4 were identified.

### Ploidy/genome size estimation

We found that 13 (76%) accessions of *D. dumetorum* were diploid (2x) and four (24%) were triploid (3x) (Table 3). The 1C genome size values for *D. dumetorum* measured here were on average 0.333 ± 0.009 pg and 0.519 ± 0.004 pg for diploids and triploids, respectively. The standard coefficient of variation (CV) of each measurement was < 5% for all runs (Additional file 4: Table S1). Comparing the data with sex, we found that diploid accessions, were 69% female and 31% were male. For triploid accessions, 75% were male and 25% female. With respect to geographic origin, all triploid accessions come from the southwestern region.

**Table 3:** Ploidy level/genome size, sex and origin of *D. dumetorum* accessions

	No. of accessions	Sex		Geographic origin				1C-values (pg)	
		Male	Female	West	Northwest	Southwest	Nigeria		
Diploid (76%)	13	4 (31%)	9 (69%)	6 (46%)	1 (8%)	4 (31%)	2 (15%)	0.333 ± 0.009	
Triploid (24%)	4	3 (75%)	1(25%)	0 (0)	0 (0)	4 (100%)	0 (0)	0.519 ± 0.004	
Diploid* (78%)	21	–	–	7 (33%)	5 (24%)	9 (43%)	0 (0)	–	
Triploid* (22%)	6	–	–	2 (33%)	1 (17%)	3 (50%)	0 (0)	–	

*Ploidy level estimated using the R package gbs2ploidy

Using the R package gbs2ploidy on accessions with known ploidy (17), we assessed the sensitivity of gbs2ploidy on our GBS data. The probability of concurrence between flow cytometry and gbs2ploidy was 35%, with 8 of 17 accessions assigned to the opposite cytotype and three (A09I, B09W, E08I) being inconclusive. The probability of correct diploid and triploid assignments was 38 and 25%, respectively. Training gbs2ploidy with reference accessions from flow cytometry on the remaining accessions (27), we found that 21 (78%) accessions were diploids and 6 (22%) triploids with the mean assignment probability of 74 and 73%, respectively. Regarding diploid accessions, seven, five and nine accessions originated from western, north-western and south-western regions, respectively. For triploids, three were from north-western, two from western and one from south-western regions. In summary, 34 accessions of *D. dumetorum* (77%) were diploid (2x) and 10 (23%) were triploid (3x). Triploid accessions originated mainly (70%) from the south-western region.

### Historical relationship between accessions

**Fig. 5:**
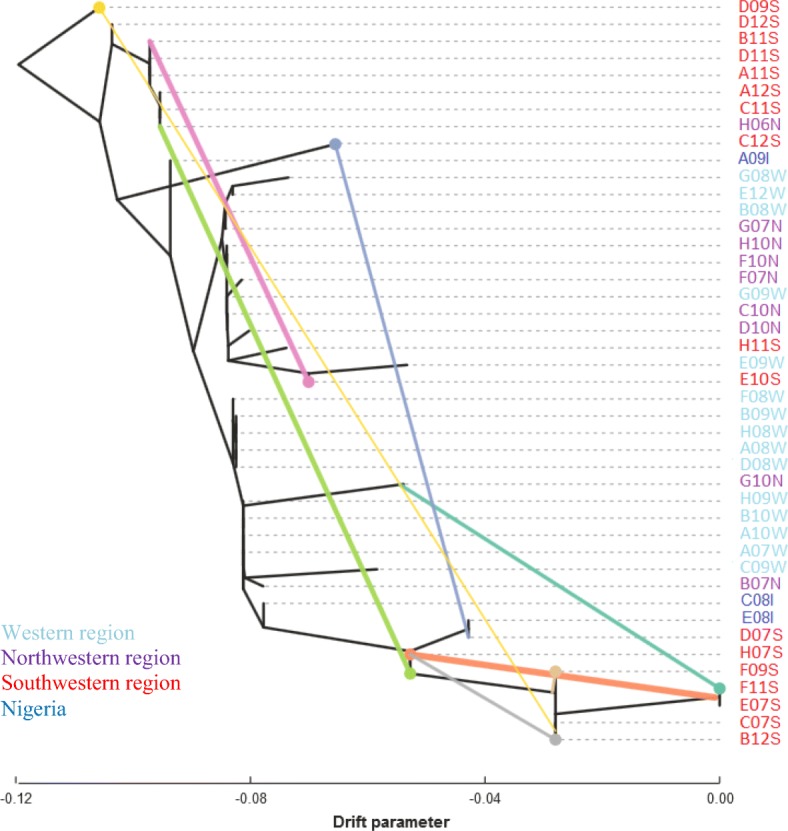
Maximum likelihood tree of the inferred gene flow within *D. dumetorum* species on 157 SNPs. The colored lines represent the possible gene flow events

We used TreeMix in order to determine splits and gene flow between *D. dumetorum* accessions. We constructed the tree allowing between no migration and ten migration events. We found eight gene flow events between *D. dumetorum* accessions (Fig. 5). Despite the likelihood for the tree with nine migration events being highest (but almost similar to eight migrations), we chose the one with eight events because the ninth migration was redundant (Additional file 5). The migration events involved eleven accessions from the south-western region and two (G10 N and H06N) from the north-western region. We did not find a migration event involving A08, which does not harden after harvest, as well accessions originating from the western region and Nigeria. C12S (2x, few root and white flesh) was possibly the result of gene flow between D07S (2x, female, few root and white flesh) and D09S (3x, male, few root and white flesh) or their ancestors; C07S (3x, male, few roots and white flesh) and E07S (2x, male, many root and yellow flesh) were possibly the result of a introgression between H06N (2x, few roots and yellow flesh) and H07S (2x, male, many roots and yellow flesh). Furthermore, allowing migrations altered the topology of the tree compared to the tree with no migrations events (Additional file 6: Figure S4).

## Discussion

Genotyping-by-sequencing is an innovative, robust and cost-effective approach allowing multiplexing individuals in one library to generate thousands to millions of SNPs across a wide range of species [51]. In our study, we identified on average 30,698 reads per accession. After filtering to avoid the effect of missing data, 5054 loci were kept for the analyses. In total, 26,325 SNPs were investigated. These numbers are similar to a previous study using the same pipeline in another non-model species [21].

The unrooted neighbor-net tree (Fig. 3) clustered *D. dumetorum* accessions into four groups: A western/north-western group, a western group, a south-western group and a mixed group. The West and North-west belong to agro-ecological zone III (Western Highlands) and the Southwest belongs to agro-ecological zone IV. This result disagrees with previous results using morphological characters [15], in which there was no clear separation of *D. dumetorum* accessions according to agro-ecological zone. However, morphological markers are subject to environmental conditions and thus provide limited genetic information. Moreover, Sonibare et al. [9] using AFLP on *D. dumetorum* accessions from three countries didn’t find a clear separation according to the area of collection. However, SNP markers are the most abundant in a genome and suitable for analysis on a wide range of genomic scales [52, 53]. In combination with high-throughput sequencing, thousands to millions of SNP generated using GBS [54] allow assessing more efficiently the genetic diversity compared to AFLP. This was already suggested by Saski et al. [24], who stated that GBS is a powerful tool for high-throughput genotyping in yam.

Our assignment test results based on STRUCTURE also separate *D. dumetorum* accessions into four populations in which three were clearly identified, the western/north-western population, the western population and the south-western population. On the contrary, MavericK revealed that *D. dumetorum* was structured into two populations in accordance with known agro-ecological zones (Additional file 2, Figure S2). However, the number of loci investigated was large (more than hundreds of loci). In this situation, the heuristic approximation implemented in STRUCTURE appears to be better [45]. Furthermore, tuber flesh color of all accessions in the western and northwestern region was yellow whereas the majority of accessions from south-western have white tuber flesh. Our results suggest that altitude and farmers-consumers preference played a role as a barrier between *D. dumetorum* populations. Indeed, AEZ 3 corresponds to western highlands covering the western and northwestern region. It is characterized by high altitude (1000–2740 m), low temperature (annual mean 19 °C) and annual rainfall of 1500 to 2000 mm. In contrast, AEZ 4 comprises mainly humid forest covering the south-western and littoral regions. It is characterized by low altitude (< 700 m except a few mountains), with an annual rainfall of 2500 to 4000 mm and a mean temperature of 26 °C [38]. All three regions of Cameroon belong to the yam belt, where the species occurs in both wild and cultivated forms. Nevertheless, its center of origin remains unknown so far excluding an explanation for the origin of the separation of populations in Cameroon. Tuber quality is an important criterion of adoption of yam varieties by farmers and consumers [55]. Thus, the difference regarding tuber flesh color in the western/north-western and south-western regions could be explained by different preferences of consumers in these regions, which also depends on yam food form. In the western and north-western regions, yam tubers are almost exclusively consumed as boiled tubers contrary to the South-west where tubers are consumed either boiled or pounded. Consumers in Cameroon probably prefer yellow tubers in the boiled and white tubers in the pounded form. Indeed, Egesi et al. [56] demonstrated that flesh color determines a general preference for boiled or pounded yam in *D. alata*. Assuming white flesh as the ancestral character state based on its predominant occurrence in other yams species, we assumed that the yellow flesh color has evolved several times (probably four times) because it is present in our four groups inferred, although a single origin with subsequent intraspecific hybridization or losses cannot be excluded. Yams with many roots have likely evolved once, in the western region probably due to environmental conditions of the highland with occasional scarcity of water. The root system has an important physiological function in nutrient and water absorption. It is well known, that several root system traits are considered to be important in maintaining plant productivity under drought stress [57]. The occurrence of mutations related to yellow flesh color and many roots on the tuber in the south-western region (mixed group) was probably caused by artificial crossing of genetically diverse accessions in the region.

The importance of gene flow within and between our four main groups in *D. dumetorum* can be seen in the high proportion of admixture. This observation could be explained by the efforts, which have been made in the past in Cameroon, especially in the South-west to improve *D. dumetorum* [7]. Indeed, genetic diversity can be increased by breeding activities [58]. Especially noteworthy is the fourth groups with all individuals assigned to it being admixed, suggesting the absence of genetically unambiguous accessions belonging to this group from Cameroon (Fig. 3). It is possibly that genetically unambiguous individuals of this group was not sampled within Cameroon or went extinct, but our preferred hypothesis is that such plants originated from Nigeria. This finding further corroborates a close relationship between *D. dumetorum* accessions from Nigeria and Cameroon. The South-west and North-west regions of Cameroon share a common border with Nigeria. Exchanges of *D. dumetorum* accessions between farmers on both side of the border are well-known, providing gene flow and interbreeding. Indeed, Sonibare et al. [9] reported that introduction of the *D. dumetorum* germplasm to Central African countries has been affected by the activities of farmers from Nigeria.

TreeMix results obtained in our study also indicate that there was more gene flow between accessions from the south-western region than in the western/north-western region. These findings support the admixture result of STRUCTURE discussed above and allow refinement of our understanding of genotypes crossed in the past. However, regarding the sample with non-postharvest hardening, we did not detect any gene flow. This suggests that the sample was not used, yet, in any breeding in Cameroon and that non-postharvest hardening appears yet to be restricted in *D. dumetorum* to Nigeria. Thus, a broader study on the genetic diversity involving samples across the distribution range of the species is needed to track the origin of this character and the ancestry of this sample.

Ploidy is another factor possibly relevant for population structure and breeding causing hybrid vigor (heterosis) and buffering of deleterious mutations. Our analysis revealed that 77% of *D. dumetorum* accessions were diploid and 23% were triploid. This result is broadly consistent with previous findings, in which 83% were diploid and 17% triploid [36] and 60% diploid and 40% triploid [37]. However, the probability of concurrence between flow cytometry and gbs2ploidy was low (35%). In fact, a limitation of the gbs2ploidy method is low coverage, especially if possible ploidy levels for the species are unknown [49]. The authors reported that this problem could be resolved by including validated reference samples with known cytotypes in the analysis as done in our study.

The association between sex and ploidy showed a predominance of triploids for male accessions and diploids for female accessions. These findings partially contradict those of Adaramola et al. [37] in which a predominance of diploid for male accessions has been reported. However, Adaramola et al. [37] outlined that a more systematic sampling method that ensures an equal number of *D. dumetorum* accession may change their results, which was the case in our study. The 1C genome size values of *D. dumetorum* accessions ranged on average from 0.33 to 0.52 pg for diploids and triploids, respectively. This supports the results of Obidiegwu et al. [36], who found that the 1C genome of five diploid and one triploid *D. dumetorum* clones ranged from 0.35 to 0.53 pg, respectively. Thus, *D. dumetorum* appears to have a very small size genome (1C-value ≤1.4 pg) following the categories of [59]. TreeMix results suggested admixture of some accessions between different ploidy levels. Triploid accessions may either be the result of a possible admixture between triploid (3x) or diploid (2x) male with diploid (2x) females, although sex of accessions H06N and C12S has not been determined. Similar results were reported in *D. alata* [60]. This suggests that the occurrence of triploid accessions in *D. dumetorum* is most likely due to the involvement of unreduced (2n) gametes in the pollen rather than the egg cell. This was confirmed by artificial crossing of triploid (3x) male and diploid (2x) female we performed in the field (Siadjeu unpublished data, Additional file 7: Figure S5). Finally, the predominant occurrence of triploid accessions in the southwestern region coincides with the more intensive breeding program in the region and may be explained by it since it is known that hybridization between genetically diverse accessions of a species may increase the number of unreduced gametes [61].

## Conclusions

In this study, we reported population structure, genetic diversity and ploidy/genome size of *D. dumetorum* in Cameroon using GBS. We demonstrated that *D. dumetorum* is structured into populations. There is a high genetic variability of *D. dumetorum* accessions in Cameroon. We revealed intraspecific hybridization and provided useful information regarding ploidy/genome size of *D. dumetorum*. All this information is relevant for conservation and a breeding program of *D. dumetorum*. However, we did not infer a firm relationship of the sample with postharvest hardening, the character most important for future breeding efforts, suggesting a broad study with respect to this character in West and Central Africa will be needed to elucidate its origin. Finally, GBS appears as an efficient powerful tool for phylogeographic studies in yams.

## Additional files

Additional file 1: **Figure S1.** Phylogenetic relationships within *D. dumetorum* based on multilocus concatenated SNP sequences alignment from GBS data of 34 diploid accessions. (PDF 259 kb)

Additional file 2: **Figure S2.** Estimates of the model evidence for K = 2:5 using TI estimator **a**) log-evidence and **b**) the evidence and Structure estimator Delta K ∆K **c**) (PDF 95 kb)

Additional file 3: **Figure S3.** STRUCTURE plot of 44 accessions of *D. dumetorum* with K = 2, 3, 5 clusters based on 6457 unlinked SNPs. (PDF 133 kb)

Additional file 4: **Table S1.** Coefficient of variation of ploidy measurements using flow cytometric and ploidy level per accessions estimated by gbs2ploidy. * Ploidy level assessed by gbs2ploidy (PDF 47 kb)

Additional file 5: Edges, weight of migrations (8 and 9) and likelihood for migrations 0:9. (TREEOUT 1 kb)

Additional file 6: **Figure S4.** Maximum likelihood tree of the inferred gene flow within *D. dumetorum* species with no gene flow events. (PDF 125 kb)

Additional file 7: **Figure S5.** Flowing and fructification of *D. dumetorum*. a) male flower, b) female flower. Bar scale = 3 cm. c) fruits, d) seeds. Bar scale = 2 cm (PDF 288 kb)

## Abbreviations

AEZ: Agro-ecological zone
AFLP: Amplified Fragment Length Polymorphism
CV: Coefficient of Variation
EDTA: Ethylenediaminetetra-acetic acid
GBS: Genotyping-By-Sequencing
MCMC: Markov Chain Monte Carlo
P: Population
PCR: Polymerase Chain Reaction
RAPD: Random Amplified Polymorphic DNA
RFLP: Restriction Fragment Length Polymorphism
SNP: Single Nucleotide Polymorphism
SSR: Single Sequence Repeats
TI: Thermodynamic Integration
