# Supplementary figures and images for: Genetic diversity and population structure of trifoliate yam (Dioscorea dumetorum Kunth) in Cameroon revealed by genotyping-by-sequencing (GBS)
Source: BMC Plant Biol. 2018 Dec 18;18:359. doi: 10.1186/s12870-018-1593-x (PMC6299658; doi:10.1186/s12870-018-1593-x)

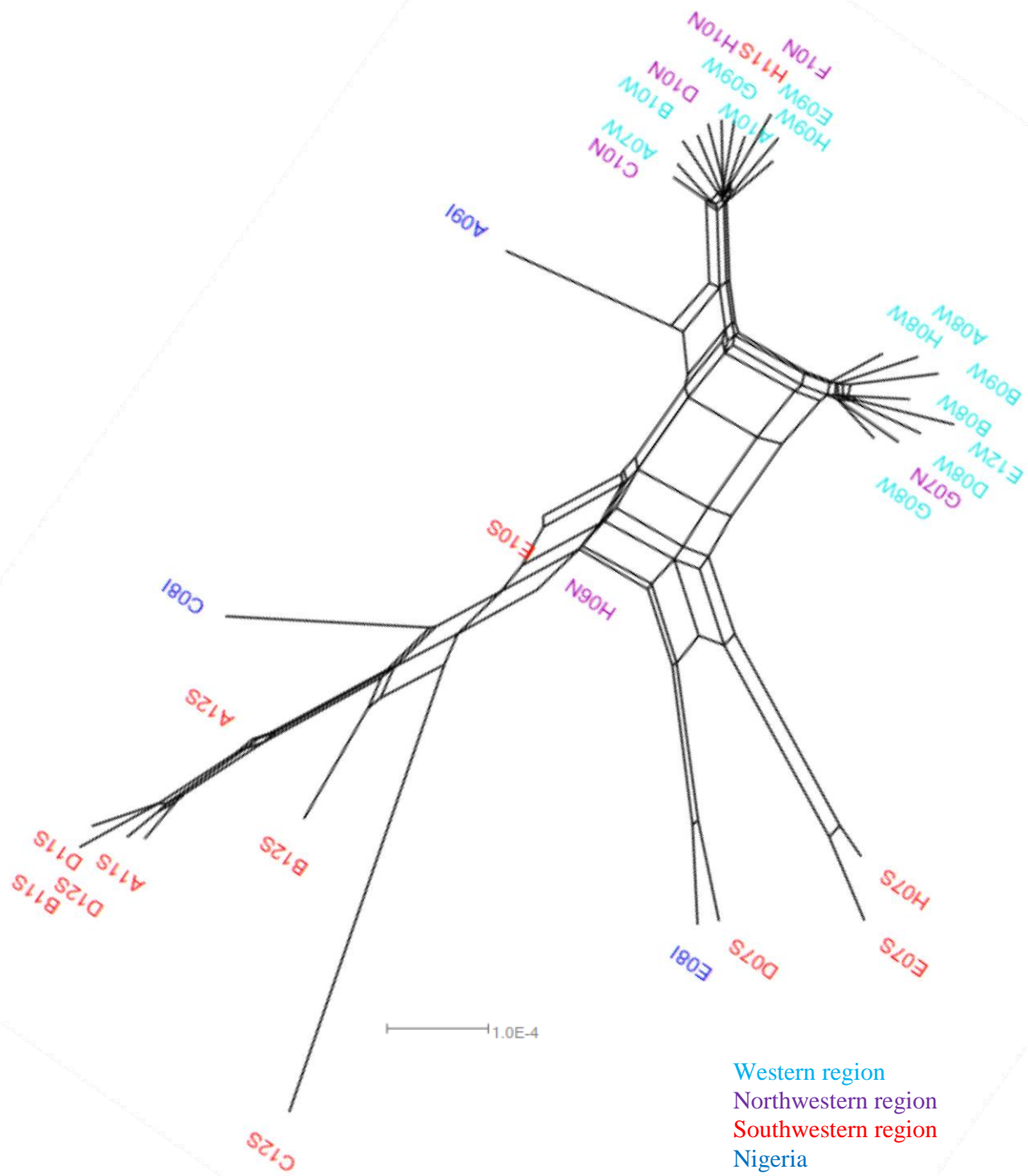

Supplement: Supplementary file 1 — Figure S1. Phylogenetic relationships within D. dumetorum based on multilocus concatenated SNP sequences alignment from GBS data of 34 diploid accessions. (PDF 259 kb) [file 12870_2018_1593_MOESM1_ESM.pdf]

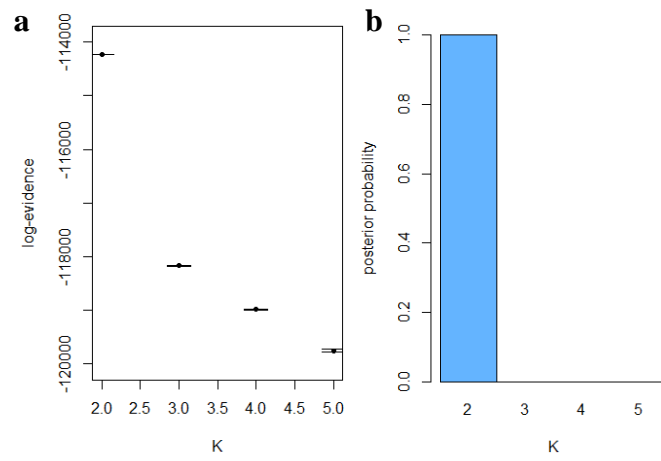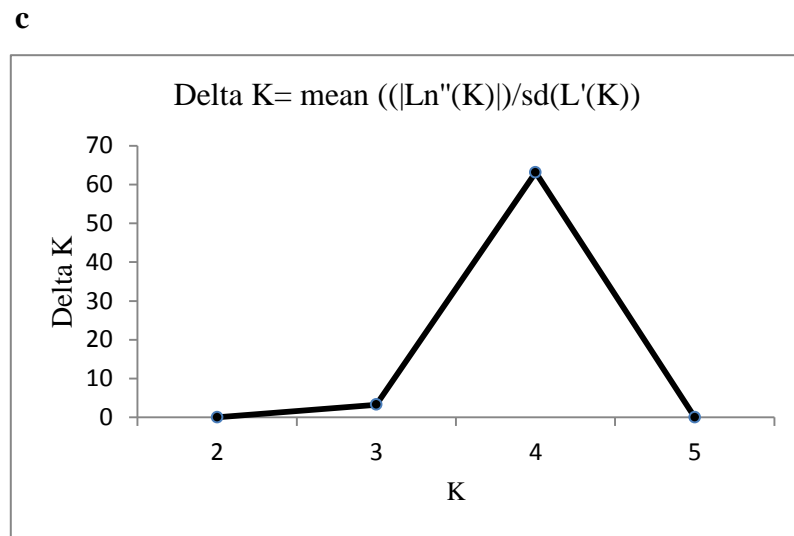

Supplement: Supplementary file 2 — Figure S2. Estimates of the model evidence for K = 2:5 using TI estimator a) log-evidence and b) the evidence and Structure estimator Delta K ∆K c) (PDF 95 kb) [file 12870_2018_1593_MOESM2_ESM.pdf]

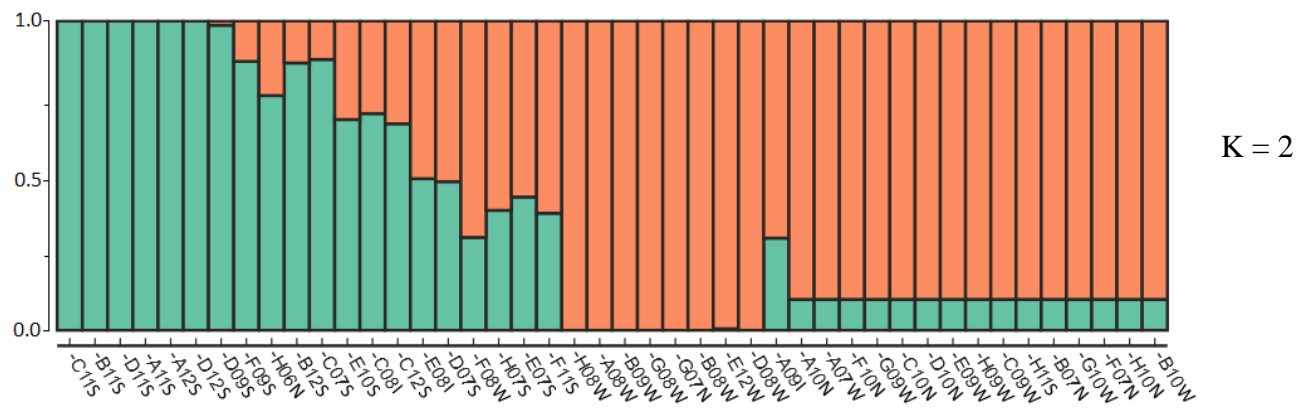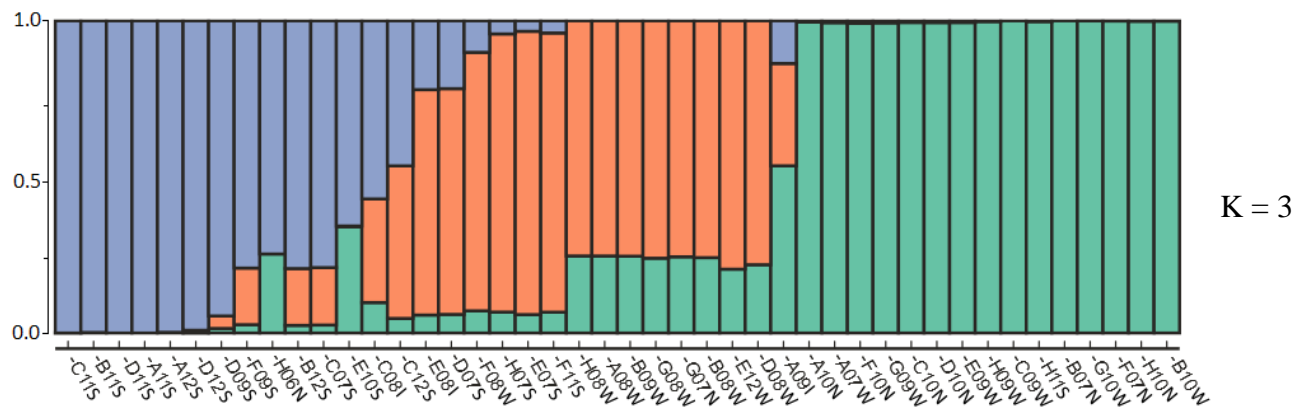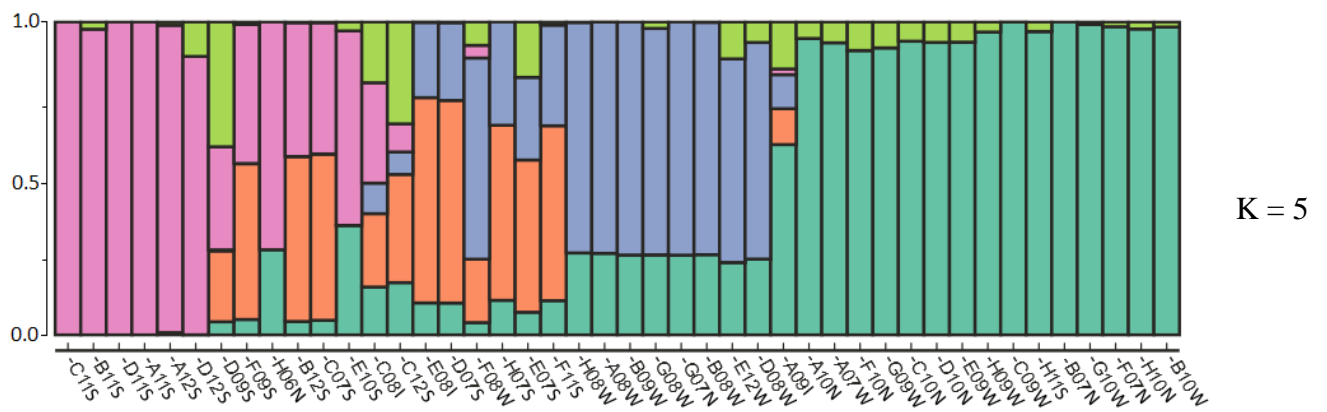

Supplement: Supplementary file 3 — Figure S3. STRUCTURE plot of 44 accessions of D. dumetorum with K = 2, 3, 5 clusters based on 6457 unlinked SNPs. (PDF 133 kb) [file 12870_2018_1593_MOESM3_ESM.pdf]

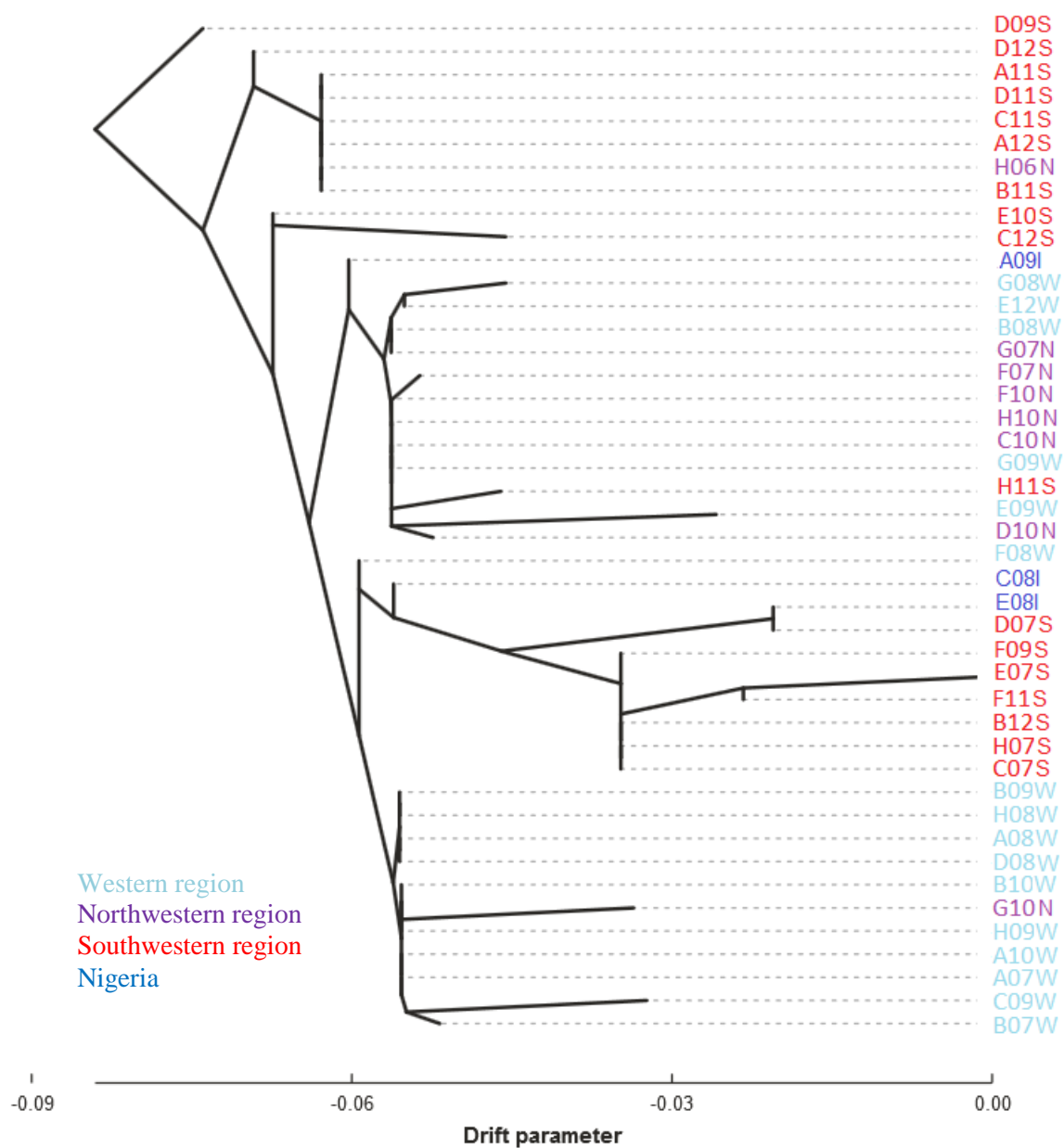

Supplement: Supplementary file 6 — Figure S4. Maximum likelihood tree of the inferred gene flow within D. dumetorum species with no gene flow events. (PDF 125 kb) [file 12870_2018_1593_MOESM6_ESM.pdf]

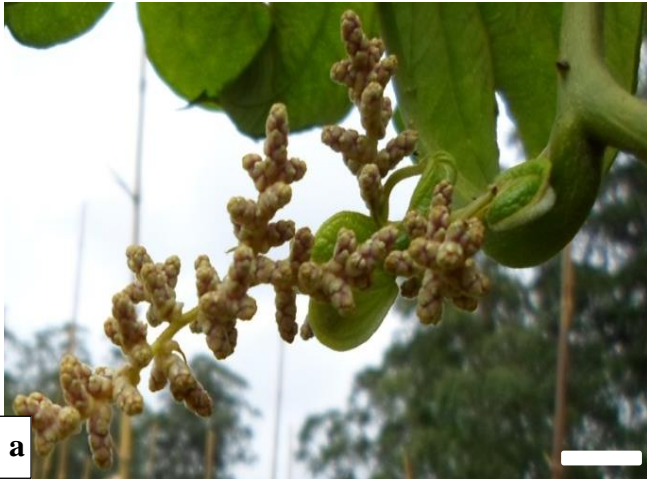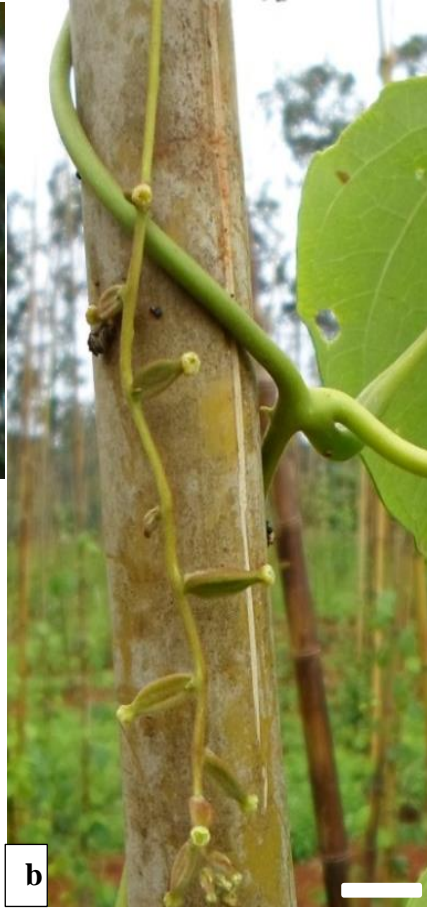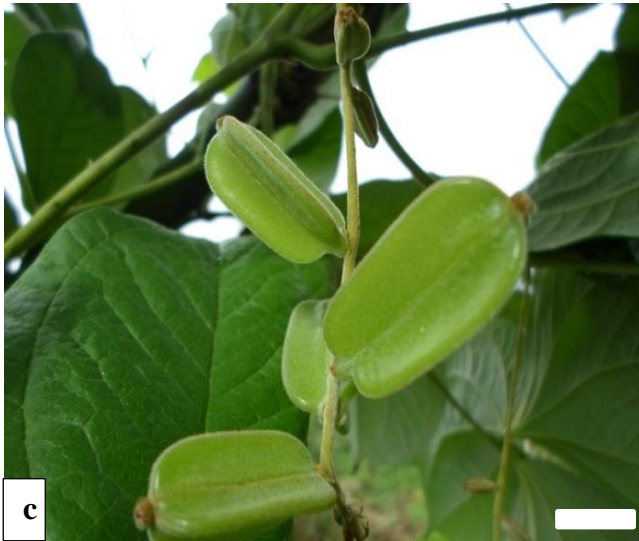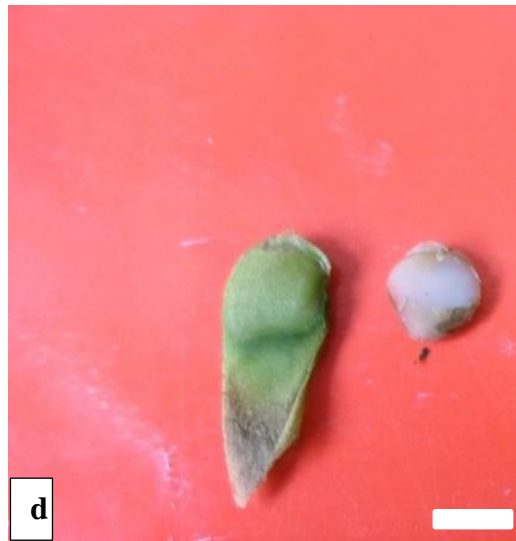

Supplement: Supplementary file 7 — Figure S5. Flowing and fructification of D. dumetorum. a) male flower, b) female flower. Bar scale = 3 cm. c) fruits, d) seeds. Bar scale = 2 cm (PDF 288 kb) [file 12870_2018_1593_MOESM7_ESM.pdf]
