# Supplementary material for: Genetic diversity and population structure of trifoliate yam (Dioscorea dumetorum Kunth) in Cameroon revealed by genotyping-by-sequencing (GBS)
Source: BMC Plant Biol. 2018 Dec 18;18:359. doi: 10.1186/s12870-018-1593-x (PMC6299658; doi:10.1186/s12870-018-1593-x)
Supplement: Supplementary file 4 — Table S1. Coefficient of variation of ploidy measurements using flow cytometric and ploidy level per accessions estimated by gbs2ploidy. * Ploidy level assessed by gbs2ploidy (PDF 47 kb) [file 12870_2018_1593_MOESM4_ESM.pdf]

Code	Origin	Sex	Ploidy level	1C-values	CV	Probability
A09I	Nigeria	Male	2x	0.329	4.52	
B08W	West	Female	2x	0.320	4.93	
B09W	West	Female	2x	0.345	4.40	
C08I	Nigeria	Male	2x	0.340	3.97	
D07S	Southwest	Female	2x	0.330	4.08	
E07S	Southwest	Male	2x	0.322	4.86	
E08I	Nigeria	Female	2x	0.336	3.89	
E12W	West	Female	2x	0.333	4.64	
G07N	Northwest	Female	2x	0.322	4.79	
G08W	West	Female	2x	0.338	4.54	
G09W	West	Female	2x	0.350	4.22	
H07S	Southwest	Male	2x	0.329	4.17	
H08W	West	Female	2x	0.333	4.50	
C07S	Soutthwest	Male	3x	0.514	4.94	
C11S	Southwest	Female	3x	0.520	3.08	
D09S	Southwest	Male	3x	0.520	4.60	
F09S	Southwest	Male	3x	0.523	3.51	
A07W*	West		2x			79.88
A08W*	West		2x			55.35
A10W*	West		2x			76.19
A11S*	Southwest		2x			77.65
A12S*	Southwest		2x			75.04
B10W*	West		2x			79.22
B11S*	Southwest		2x			60.45
B12S*	Southwest		2x			70.51
C10N*	Northwest		2x			73.86
C12S*	Southwest		2x			75.72
D08W*	West		2x			61.9
D10N*	Northwest		2x			83.35
D11S*	Southwest		2x			71.07
D12S*	Southwest		2x			51.05
E09W*	West		2x			74.7
E10S*	Southwest		2x			71.55
F10N*	Northwest		2x			84.42
H06N*	Northwest		2x			84.78
H09W*	West		2x			79.1
H10N*	Northwest		2x			83.66
H11S*	Southwest		2x			79.89
B07N*	Northwest		3x			88.51
C09W*	West		3x			83.71
F07N*	Northwest		3x			73.83
F08W*	West		3x			55.14
F11S*	Southwest		3x			58.61
G10N*	Northwest		3x			80.17
